# Evaluation of Antioxidant, Free Radical Scavenging, and Antimicrobial Activity of *Quercus incana* Roxb.

**DOI:** 10.3389/fphar.2015.00277

**Published:** 2015-11-23

**Authors:** Rizwana Sarwar, Umar Farooq, Ajmal Khan, Sadia Naz, Sara Khan, Afsar Khan, Abdur Rauf, Haji Bahadar, Reaz Uddin

**Affiliations:** ^1^Department of Chemistry, COMSATS Institute of Information TechnologyAbbottabad, Pakistan; ^2^Department of Geology, University of SwabiAnbar, Pakistan; ^3^Department of Pharmacy, Kohat University of Science and TechnologyKohat, Pakistan; ^4^Dr. Panjwani Center for Molecular Medicine and Drug Research, International Center for Chemical and Biological Sciences, University of KarachiKarachi, Pakistan

**Keywords:** *Quercus incana* Roxb., antimicrobial, free radical scavenging, antioxidant activity, oxidative stress

## Abstract

Considering the indigenous utilization of *Quercus incana* Roxb., the present study deals with the investigation of antioxidant, free radical scavenging activity, total phenolic content, and antimicrobial activity of *Q. incana* Roxb. *In vitro* antioxidant activity of the plant fractions were determined by 1,1-diphenyl-2-picrylhydrazyl and nitric oxide scavenging method. Total phenolic contents were determined by gallic acid equivalent and antimicrobial activities were determined by agar well diffusion method. It was observed that *Q. incana* Roxb. showed significant antibacterial activity against Gram-positive and Gram-negative bacteria. *n*-Butanol fraction showed maximum activity against *Micrococcus leuteus* with 19 mm zone of inhibition. *n*-Butanol fraction of *Q. incana* Roxb. showed immense antifungal activity against *Aspergillus niger* (32 mm ± 0.55) and *A. flavus* (28 mm ± 0.45). Similarly *n*-butanol fraction showed relatively good antioxidant activity with IC_50_ value of 55.4 ± 0.21 μg/mL. The NO scavenging activity of ethyl acetate fraction (IC_50_ = 23.21 ± 0.31 μg/mL) was fairly good compared to other fractions. The current study of *Q. incana* Roxb. suggests the presences of synergetic action of some biological active compounds that may be present in the leaves of medicinal plant. Further studies are needed to better characterize the important active constituents responsible for the antimicrobial, antioxidant and free radical scavenging activity.

## Introduction

Oxidative damage is a major source of many illnesses; as free radicals, and reactive oxygen species (ROS) attacks cell macromolecules. Antioxidants play key role in preventing cell being injured by ROS by counter acting these free radicals (Lai et al., 2001). Antioxidants derived from plants provide protection to cell by scavenging free oxygen radical through offsetting ROS. This has been made possible due to the presence of certain bioactive substances, such as phenolic compounds, flavonoids, and essential oils, rendering plants with antioxidant activity (Liu, 2003). Nitric oxide (NO) acts as neurotransmitter through exerting their effect on different body operations, such as neurotransmission, synaptic plasticity, vasodilation, and CNS memory (Shibuki and Okada, 1991; Bredt and Snyder, 1994). Besides key role of NO in facilitating normal function, it has been observed that NO has been associated with pathophysiologic states like neurodegenerative and Alzheimer’s disease. Excessive release of NO in the body can cause DNA fragmentation, cell damage, and neuronal cell death (Moncada et al., 1991; Dawson et al., 1992). Plants can play crucial role in reducing amount of NO through their efficient NO scavenging activity.

In order to amplify the data corroborating potent plant that may be exploited as antimicrobial and antioxidant agent, the *Quercus incana* Roxb. was selected to be assessed for possible medicinal action. *Q. incana* Roxb. belongs to family *Fagaceae*, represented by 8 genera and 900 species, abundantly found in the temperate regions of the world. In Pakistan, this family is represented by two genera namely *Castana* and *Quercus*. *Quercus* is represented by six species in Pakistan most of which are wild, distributed in northern temperate regions. The wood of this genus is durable, tough and is widely used for shipbuilding, flooring, furniture, railroad ties, barrels, tool handles, etc. (Adonizio et al., 2006). Due to high tannin content in *Quercus* species, it is used in tanning leather. The barrels made from *Quercus* wood are used to hoard wine and it also impart special flavor to wine. As far as its medicinal characteristics are concerned, this genus has been found to possess antioxidant, antifungal, antibacterial, and anticancer activities (Ge et al., 2008).

In folk medicine this genus is especially significant as hemostatic, in the treatment of gastrointestinal disorders (Kaur et al., 2004), inflammations of the oral, genital, and anal mucosa and externally against inflammation of the skin (Konig et al., 1994). Polar fractions of the leaves, bark, wood, and galls have shown antibacterial and anti-inflammatory activities (Ito et al., 2002; Hamid et al., 2005; Berahou et al., 2007) that explain their ethno-pharmacological uses. The ethanolic extract of *Q. leucotrichophora* exhibited a potent antimicrobial activity against *Staphylococcus aureus, Pseudomonas auroginosa*, *Bacillus subtilis*, and *Escherichia coli* (Sati et al., 2011).

Analysis on physiochemical constituent of this genus has shown that plants belonging to genus *Quercus* possess a rich load of lignins, hydrolysable tannins, ellagitannins, flavano-ellagitannins, catechins, flavan and proanthocyanidin glycosides, flavonoids and simple phenols, and proanthocyanidin glycosides (Nonaka et al., 1985; Karioti et al., 2009).

*Quercus incana* Roxb. was used as astringent (Hussain and Ghani, 2008), antidiarrheal, diuretic, and to treat asthma (Haq et al., 2011), antipyretic, anti-rheumatism, wound healing, immature abscesses, antidiabetic, and anti-arthritic (Shinwari and Khan, 2000). Considering the medicinal importance of genus *Quercus*, the *Q. incana* Roxb. was selected for the possible antimicrobial, antioxidant activity. and NO scavenging activity.

## Materials and Methods

### Plant

Leaves of *Q. incana* Roxb. were collected from District Abbottabad in May 2011. It was shade dried (16 kg) and soaked in methanol, then extracted on rotary evaporator. Crude extract (620 g) obtained as a result of extraction was partitioned between *n*-hexane (160 g), chloroform (138 g), ethyl acetate (75 g), *n*-butanol (105 g), and aqueous fraction (110 g). Fractions were subjected to antioxidant, free RSA, and antimicrobial screening by using different methods. Total phenolic content of fractions was also calculated.

### Antioxidant Activity

Free RSA of all fractions of plant were measured by DPPH method (Choudhary et al., 2008). 100 mM concentrated solution of DPPH was prepared in high-performance liquid chromatography (HPLC) grade methanol and plant fractions were dissolved in dimethyl sulfoxide (DMSO). Plant fractions were mixed with DPPH and allowed to react for half an hour at 37°C. In this assay two standard *n*-Propyl gallate and 3-*t*-butyl-4-hydroxyanisole were used. Five dilutions for each fraction along with standard were tested and experiment was repeated triplicate. After incubation absorbance was measured using microplate reader (Bio-Tek Elx800 TM, Instruments, Inc., USA). Percent RSA (% RSA) of samples was determined, while DMSO used as control group using the following formula:

$$\%RSA=100-\frac{\left[Absorbance\,\;of\,\;test\,\;compounds\right]}{Absorbance\,\;of\,\;control}\times 100.$$

### NO Scavenging Activity

Sodium nitroprusside was used for generation of NO and it was measured by the Griess reagent (1% sulphanilamide, 0.1% naphthylethylenediamine dichloride (NED), and 3% phosphoric acid). SNP spontaneously generates NO in aqueous solution at physiological pH (Marcocci et al., 1994) results in production of nitrite ions by its interaction with oxygen, whose estimation is done by Griess reagent. Scavengers of NO compete with oxygen leading to reduced production of NO. Different concentrations (100–1000 μg/mL) of plant fractions dissolved in ethanol and water was mixed with SNP (10 mM) in phosphate buffer saline (PBS) and incubated at 25°C for 3 h. The samples were then reacted with griess reagent, and absorbance was recorded at 546 nm of chromophore formed as result of diazotization of nitrite with sulphanilamide, and subsequent coupling with NED was done using microplate reader and compared to positive control which in this case was ascorbic acid treated in same way to Griess reagent. The ethanol was used as control using the following formula:

$$Nitric\,\;oxide\,\;scavenged\left(\%\right)=\frac{\left(A_{control}-A_{test}\right)}{A_{control}}\times 100.$$

### Total Phenolic Contents of Fractions

Total phenolic content of extract was determined by spectroscopic method using Folin–Ciocalteu reagent (diluted 10-fold) and gallic acid as standard (Osawa and Namiki, 1981). Ethanol solution of gallic acid (1 mL; 0.025–0.400 mg/mL) was mixed with 5 mL Folin–Ciocalteu reagent (diluted tenfold) and sodium carbonate (4 mL, 0.7 M) to give calibration curve. Standard curve was drawn after measuring absorbance at 765 nm. 1 mL of plant crude fraction (5 g/L) was also mixed with the above reagents, and left for 30 min. The total phenolic content was analyzed against gallic acid standard curve by taking absorbance. Readings were taken in triplicate. The total phenolic of all fractions in GAE were calculated by the following formula:

$$T=\frac{CV}{M}$$

Where T is the total phenolic contents, mg/g of fraction, in GAE; *C* is the concentration of gallic acid established from the calibration curve, mg/mL; *V* is the volume of the fraction, mL; and *M* is the weight of the fraction (g).

### Antibacterial Activity

Each fractions of *Q. incana* Roxb. were tested separately, employing agar well diffusion method (Harit et al., 2013; Bag and Chattopadhyay, 2015; Zhou et al., 2015). The medium was sterilized by autoclaving at 120°C and 20 ml of the agar medium was aseptically transferred into each sterilized Petri plate, which was solidified at room temperature. Bacterial strains were spread on agar plates with sterile cotton swab. A well of 6 mm diameter was made using a sterile cork borer. All fractions (3 mg) were dissolved in 1 mL of 20% DMSO solution. The 20 μL of standard drug and fractions of the plant were poured in 6 mm diameter well. The assay plates were incubated at 37°C for 24 h. The standard disk with ciprofloxacin (50 μg per disk) was used as a positive control for antibacterial activity and DMSO was used as negative control. Zone of inhibition was measured in mm and displayed in graph.

### Antifungal Activity

The antifungal activity was done by disk diffusion method (Bobbarala et al., 2009). Sabouraud dextrose agar plates were inoculated with each fungal strain by point inoculation. The filter paper disk (6 mm in diameter) impregnated with 1 mg mL^-1^ of the fractions were placed on seeded plate. DMSO was used as solvent and was completely evaporated before applying to Petri plates. Blank disk impregnated with DMSO was used as negative control and nystatin was used as positive control. The activity was determined after 72 hrs of incubation at 28°C and the diameters of the inhibition zones were measured in mm.

## Results And Discussion

### Antioxidants

**FIGURE 1:**
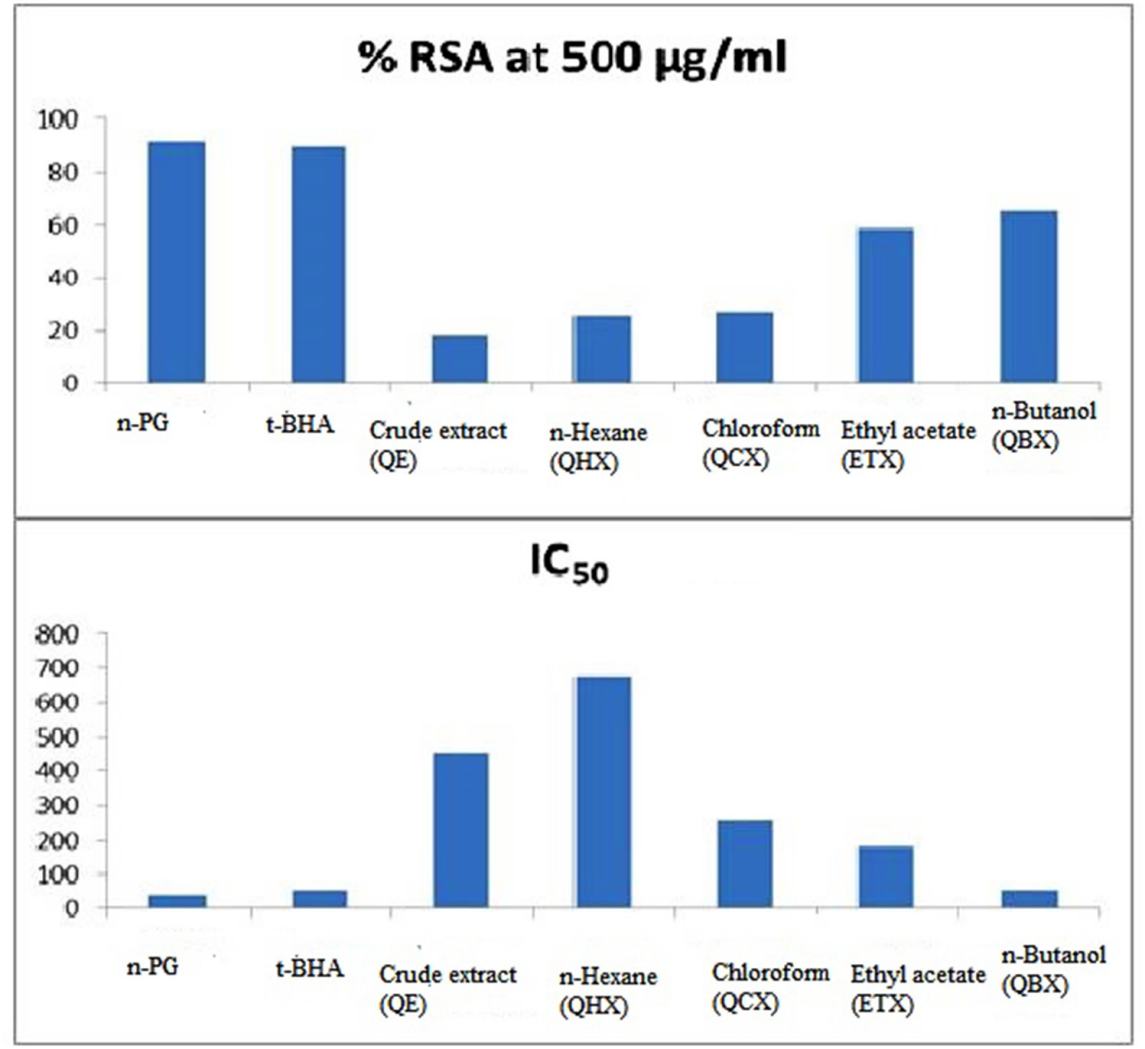
**Antioxidant activity in % RSA and IC_50_**.

The results of the free radical scavenging potential of fractions of *Q. incana* Roxb. was tested by DPPH method was presented in **Figure 1**. Antioxidant reacts with DPPH, which is a nitrogen-centered radical with a characteristic absorption at 517 nm and converts it to DPPH, due to its hydrogen accepting ability at a very rapid rate (Yamaguchi et al., 1998).

The degree of discoloration indicates the scavenging potentials of the antioxidant present in fractions. Result showed that highest RSA was observed in *n*-butanol and ethyl acetate with % inhibition of 64.85 and 58.89%, respectively, while least in *n*-hexane fraction with 26.02% (**Figure 1**). Greater % inhibition of *n*- butanol and ethyl acetate fractions were due to the presence of high polyphenolics, flavonoids, and tannins in the extracts. IC_50_ values were calculated using regression equation that showed inverse relationship between IC_50_ and percentage scavenging thus strongest DPPH radical scavenging was observed in *n*-butanol and ethyl acetate fraction with IC_50_ values of 55.4 ± 0.21 and 182.3 ± 0.85 μg/mL, respectively, while lowest was in *n*-hexane 677.8 ± 0.67 μg/mL. Standards used in this experiment, i.e., *n*-propyl gallate and 3-t-butyl-4-hydroxyanisole showed % RSA at 91.72% and 89.56% with IC_50_ value of 40.13 ± 0.74 and 58.76 ± 0.45 μg/mL, respectively (**Table 1**).

**Table 1:** Antioxidant potential of *Querus incana* Roxb.

Plant extracts	% RSA at 500 μg/ml	IC_50_ (μg/ml)
*n*-PG (Standard)	91.72	40.13 ± 0.74
*t*-BHA (Standard)	89.56	58.76 ± 0.45
Crude extract (QE)	18.41	456.4 ± 0.27
*n*-Hexane (QHX)	26.02	677.8 ± 0.67
Chloroform (QCX)	27.24	258.8 ± 1.03
Ethyl acetate (ETX)	58.89	182.2 ± 0.85
*n*-Butanol (QBX)	64.85	55.4 ± 0.21

### NO Scavenging Activity

**FIGURE 2:**
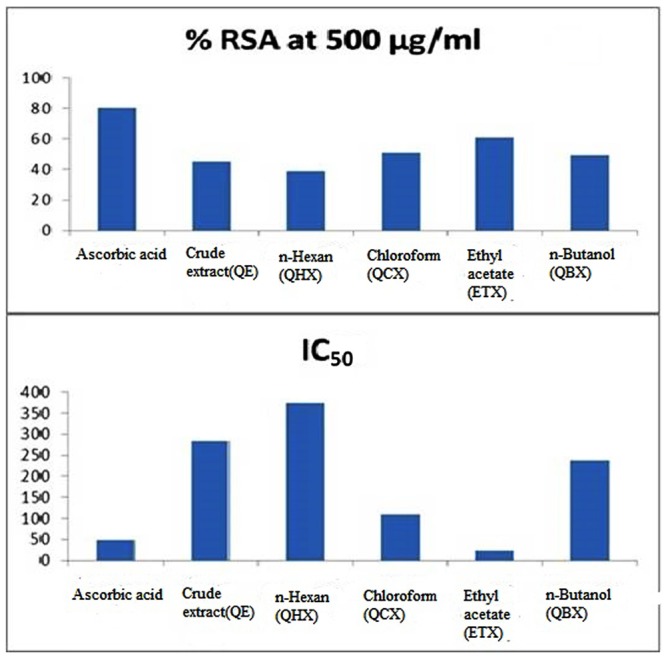
**NO activity in % RSA and IC_50_ values**.

Nitric oxide is a potent pleiotropic mediator of physiological processes such as smooth muscle relaxation, inhibition of platelet aggregation, neuronal signaling, and regulation of cell mediated toxicity. It is a diffusible free radical which plays many important roles as an effector molecule in diverse biological systems (vasodilation, neuronal messenger, and antimicrobial and antitumor activities). Ascorbic acid used as the standard for NO radical scavenger in this analysis showed maximum activity with percentage RSA (%RSA) of 80.42% with IC_50_ value 48.88 ± 0.74 μg/mL (**Table 2**). Among all fractions ethyl acetate showed maximum RSA of 60.88% while *n*-hexane and crude fraction showed least activity with RSA of 38.84 and 45.02%, respectively. The IC_50_ values calculated for each fraction of *Q. incana* Roxb. revealed that ethyl acetate, chloroform and *n*-butanol were highly active with IC_50_ value 23.21 ± 0.31, 108.63 ± 0.78, and 238.17 ± 0.16 μg/mL, respectively, while *n*-hexane showed least activity with IC_50_ value of 374.27 ± 0.28 μg/mL (**Figure 2**).

**Table 2:** NO scavenging activity of *Querus incana* Roxb.

Plant extracts	% RSA at 500 μg/ml	IC_50_ (μg/ml)
Ascorboic acid (Standard)	80.42	48.88 ± 0.74
QE	45.02	282.87 ± 0.08
QHX	38.84	374.27 ± 0.28
QCX	50.94	108.63 ± 0.78
ETX	60.88	23.21 ± 0.31
QBX	49.17	238.17 ± 0.16

*Each value in the table was obtained by calculating the average of three experiments ±SD.*

### Total Phenolic Content

Phenolic compounds, as secondary metabolites, are considered as the main actors for the antioxidant capacity of plants and have also many benefits on human health, as free radical scavenger. Phenolic compounds are also known as powerful chain breaking antioxidants. It is hard to estimate the real content of the phenolic compounds, due to the fact that the phenolic content are largely influenced by many factors, such as biotic and abiotic stress, senescence, cultivar, tissue, harvesting time, post-harvest treatment, and also extraction techniques. As reported in **Table 3**, result showed the significant total phenolic content in fractions of *Q. incana* Roxb. was observed.

**Table 3:** Total phenolic content of *Q. incana* Roxb.

Plant extracts	Total phenol (mg/g)
QE	165 ± 6
QHX	43 ± 4
QCX	56 ± 7
ETX	65 ± 7
QBX	89 ± 3

*Each value in the table was obtained by calculating the average of three experiments ±SD.*

*n*-Butanol fraction was found as the highest phenolic contents 89 ± 3 mg/g fraction as compared to other fractions. The high concentration of polyphenolic in the *n*-butanol fraction may be due to purification and concentration of phenolic, throughout the fractionation procedure and it is probably responsible for its high free RSA. *n*-Hexane fraction has least total phenolic content 43 ± 4 mg/g as compared to other fractions. Different investigations of qualitative composition of plant extracts revealed, that high concentrations of phenols in the plant extracts obtained by using polar solvents (Canadanovic-Brunet et al., 2008). Therefore, the phenolic content of plant may contribute directly to their antioxidant action (Tosun et al., 2009).

### Antibacterial

**FIGURE 3:**
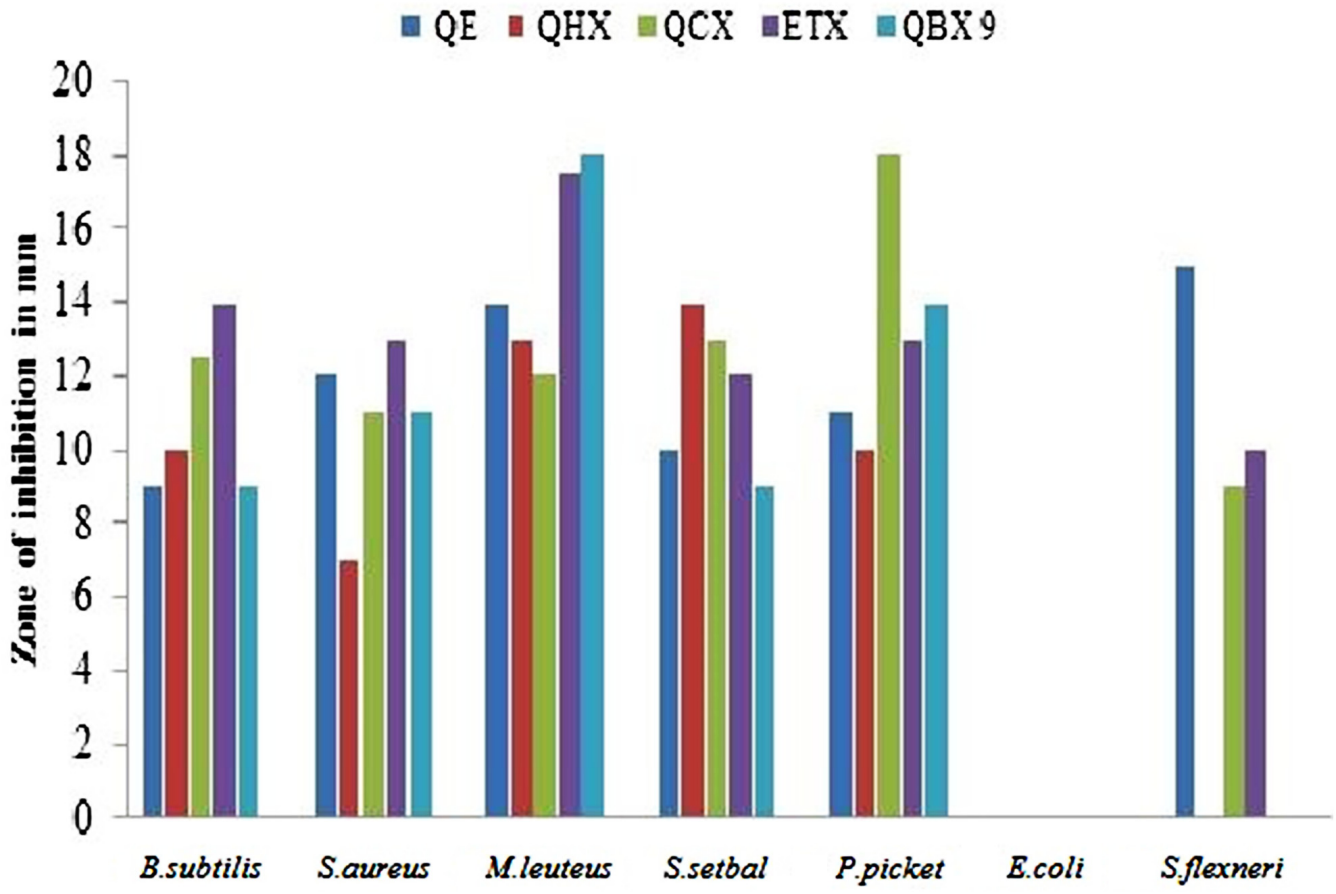
**Antibacterial activity of fractions against bacterial strains**. *B. subtilis*, *Bacillus subtilis*; *S. aureus*, *Staphylococcus aureus*; *M. leuteus*, *Micrococcus leuteus*; *S. setuba l*, *Salmonella setubal*; *P. pickettii*, *Pseudomonas pickettii*; *E. coli*, *Escherichia coli*; *S. flexneri*, *Sigella flexneri*.

The secondary metabolites produced by medicinal plants constitute a source of bioactive substances and nowadays scientific interest has increased due to the search for new drugs of plant origin. The antibacterial activity of *Q. incana* Roxb. was shown in **Table 4**. In antibacterial activity, the *n*-butanol fraction was found significantly active against *M. leuteus*, Salmonella *setubal* and *Pseudomonas pickettii* bacterial strains. Ethyl acetate fraction was active against *M. leuteus* as compared to other strains (**Figure 3**). Chloroform fraction showed good activity against *P. pickettii* and moderately active against rest of strains. Similarly *n*-hexane and *n*-butanol fractions were active against all strain except *Sigella flexneri*. All fractions were found inactive against *E. coli* (**Table 5**).

**Table 4:** Minimum inhibitory concentration (MIC) values of *Q. incana* Roxb. measured at different concentrations (mg/ml).

	Pathogenic bacteria						
Plant extracts	*Bacillus subtilis*	*Staphylococcus aureus*	*Micrococcus leuteus*	*Salmonella setubal*	*Pseudomonas pickettii*	*Escherichia coli*	*Sigella flexneri*
Ciprofloxacin (Standard)	0.75	0.187	0.75	0.187	1.50	-	0.375
QE	0.187	1.50	1.50	0.187	1.50	-	1.50
QHX	1.50	0.75	0.75	0.375	1.50	-	-
QCX	0.75	1.50	0.187	0.375	0.187	-	0.75
ETX	0.187	0.187	0.75	0.187	0.375	-	1.50
QBX	1.50	1.50	0.375	1.50	0.75	-	-

*Each value in the table was obtained by calculating the average of three experiments.*

**Table 5:** Minimum inhibitory concentration values Antifungal activity of extract measured at different concentrations (mg/ml).

Plant extracts	Pathogenic fungi	
	*A. flavus*	*A. niger*
QE	0.187	1.5
QHX	1.50	0.375
QCX	0.75	0.187
ETX	-	1.5
QBX	0.75	1.50
Nystatin (standard)	0.375	1.50

*Each value in the table was obtained by calculating the average of three experiments.*

The activities detected in the fractions of the *Q. incana* Roxb. suggest a synergistic action of some compounds, as well as the presence of other bioactive that are responsible for antibacterial activity.

### Antifungal Activity

Reported literature showed that some species of genus *Quercus* showed immense antifungal activity against pathogenic strains (Serit et al., 1991). In current study, as shown in **Table 6**, *n*-butanol fraction showed promising activity against both strains (*Aspergillus flavus* and *A. niger*) while other fractions showed moderate activity against pathogenic fungal strains. Ethyl acetate fraction was inactive against *A. flavus. Quercus* species are famous for the presence of tannins exhibiting good antimicrobial activity (Cowan, 1999; Cushnie and Lamb, 2005).

**Table 6:** Antifungal activity of *Q. incana* Roxb. (3 mg/ml, zone of inhibition in mm ± *SD*).

Plant extracts	Pathogenic fungi	
	*Aspergillus flavus*	*Aspergillus niger*
QE	19 mm ± 0.41	18 mm ± 0.85
QHX	16 mm ± 0.26	19 mm ± 0.41
QCX	12 mm ± 0.28	14 mm ± 0.66
ETX	–	25 mm ± 0.75
QBX	28 mm ± 0.45	32 mm ± 0.55
Nystatin (standard)	16 mm ± 0.92	23 mm ± 0.41

*Each value in the table was obtained by calculating the average of three experiments.*

## Conclusion

The preliminary study carried out with *Q. incana* Roxb. showing its antimicrobial activity, total phenolic content, antioxidant, and NO scavenging activities encouraged us to continue chemical investigations in order to identify the active principles of this plant. The activities of extract and fractions of *Q. incana* Roxb. suggest the presences of synergetic action of some biological active compounds that may be present in the leaves of medicinal plant. The present studies will be provided an important clue for bio-assay guided isolation of new bioactive from this plant. Further studies are needed to better characterize the important active constituents responsible for the antimicrobial, antioxidant and free RSA.

## Conflict of Interest Statement

The authors declare that the research was conducted in the absence of any commercial or financial relationships that could be construed as a potential conflict of interest.

## Abbreviations

DPPH: 1,1-diphenyl-2-picrylhydrazyl
GAE: gallic acid equivalent
RSA: radical scavenging activity
SNP: sodium nitroprusside
